# Knowledge, Practices, and Perceptions of the Healthcare Providers on Cervical Cancer Screening Among HIV-Positive Women at Lira Regional Referral Hospital, Lira City

**DOI:** 10.1155/bmri/1156001

**Published:** 2025-04-24

**Authors:** Florence Layet, Tom Murungi, Nasser Ashaba, Renniter Mirembe Nakayita, Eustes Kigongo, Marc Sam Opollo

**Affiliations:** ^1^Department of Community Health, Faculty of Public Health, Lira University, Lira City, Uganda; ^2^Department of Midwifery, Faculty of Nursing and Midwifery, Lira University, Lira City, Uganda; ^3^Department of Environmental Health and Disease Control, Faculty of Public Health, Lira University, Lira City, Uganda

**Keywords:** cervical cancer screening, healthcare providers, knowledge, perceptions, practices

## Abstract

**Background:** Cervical cancer remains a global burden and is by far one of the major causes of premature death among women of reproductive age. We explored the knowledge, practices, and perceptions of healthcare providers on cervical cancer screening (CCS) among HIV-positive women aged 18–49 years in Lira City.

**Methods:** In June 2023, a qualitative cross-sectional study was conducted among healthcare providers at Lira Regional Referral Hospital (LRRH), Lira City, Northern Uganda. Key informant interviews (KIIs) were used to collect data among purposively selected healthcare providers. Interviews were audio-recorded, transcribed verbatim, and coded using the NVivo (QSR International) software. Thematic content analysis was used in data analysis.

**Results:** The study found that participants had good knowledge about cervical cancer and screening. They provided health education and vaccination for eligible girls and screened HIV-positive women for cervical cancer. Long waiting hours, long distances to healthcare facilities, and negative attitudes of some health providers were reported as barriers to CCS utilization. However, increased awareness, the presence of signs/symptoms, and the desire to maintain optimal health facilitated CCS utilization among HIV-positive women.

**Conclusion:** Whereas the participants' knowledge and perceptions about CCS were good, their practices towards CCS among HIV-positive women were suboptimal. Continuous education of healthcare providers, decentralization of CCS, and routine and ongoing health education initiatives are pivotal in improving CCS practices among HIV-positive women.

## 1. Introduction

Cervical cancer remains a global burden and is by far one of the major causes of premature death among women of reproductive age [1, 2]. Low- and middle-income countries bear the highest burden of cervical cancer incidences and deaths among women [2]. Sub-Saharan Africa, specifically Eastern and Southern Africa, contributes the highest cervical cancer deaths and incidences as well as the highest burden of high-risk human papillomavirus (HPV) [1, 3, 4]. Infection with the HPV is responsible for the majority of cervical cancers, with HPV types 16 and 18 causing about 70% of cervical cancers [5]. However, women infected with HIV are at higher risk of developing cancer of the cervix [6], and the disease is frequent among those aged 18–49 years [7]. Annually, about 6959 new cervical cancer cases are diagnosed, with about 4607 cervical cancer deaths among women in Uganda [8].

Nearly all cervical cancers can be prevented through HPV vaccination of all eligible girls, HPV testing, routine cervical cancer screening, and early initiation of treatment for precancerous lesions [9, 10]. To reduce cervical cancer incidences and deaths, the World Health Organization (WHO) recommends regular cervical cancer screening for 70% of women and treatment for 90% of those with precancer lesions [11]. Furthermore, frequent cervical cancer screening among women living with HIV from 25 years of age is recommended [5]. Uganda recommends annual cervical cancer screening for women living with HIV [12, 13]. Unfortunately, screening among women living with HIV is still low and affected by both systematic, structural, and contextual factors [14, 15].

Healthcare providers, especially nurses, gynaecologists, medical officers, and midwives in Uganda are the primary providers of cervical cancer screening services [13]. They have more opportunities to counsel HIV-positive women on CCS because they spend more quality time with them [13, 16]. They are crucial in preventing cervical cancer by knowing screening guidelines, risk factors, screening technologies, interpretation of results, follow-up strategies, and patient counselling [16]. Thus, it is of utmost importance that healthcare providers have better practices and adequate knowledge towards cervical cancer screening. Their expertise is vital in detecting cervical abnormalities early and managing them effectively, which ultimately reduces the incidence of and mortality associated with this disease. Literature suggests that the knowledge of healthcare providers on cervical cancer screening is suboptimal [17–19], which sabotages the global targets for cervical cancer mitigation. However, evidence from some studies shows that healthcare providers are knowledgeable and are significantly contributing to cervical cancer screening [20–23].

Cervical cancer prevention measures in Lira district, such as HPV vaccination for young girls and cervical cancer screening, have been markedly suboptimal [24–26] despite the existing provider-led interventions [12]. Notably, little is known about the knowledge, practices, and perceptions of health providers towards CCS among HIV-positive women in Northern Uganda. Moreover, it is recommended that the perspectives of the healthcare providers be explored qualitatively according to a previous study in Uganda [27]. This study provides valuable insights into the issue and underlines the need for action to improve cervical cancer prevention and management in Lira City and similar settings. It is against this background that our study explored the knowledge, practices, and perceptions of healthcare providers on cervical cancer screening among HIV-positive women in Lira City.

## 2. Materials and Methods

### 2.1. Study Design and Setting

This was a cross-sectional qualitative study that used thematic analysis to identify the knowledge, practices, and perceptions of healthcare providers on cervical cancer screening among HIV-positive women in Lira City. Since the primary objective was to gain an in-depth understanding of how healthcare providers perceive CCS among HIV-positive women, qualitative methods such as key informant interviews (KIIs) provided the rich, detailed data necessary. KIIs were used to collect data among healthcare providers at Lira Regional Referral Hospital (LRRH) who were purposively selected based on (I) the Department/unit where they worked and (II) Cadre. The study used an explanatory model to investigate why HIV-positive women do not use CCS services and to understand the knowledge and practices of healthcare providers on CCS among HIV-positive women.

The study was conducted among healthcare providers at LRRH, Lira City, Northern Uganda, in June 2023. Routine cervical cancer screening services at LRRH are provided at the cervical cancer unit at a free cost, and the clinic has 5 working days a week. HIV-positive women requiring CCS services from the ART clinic, family planning unit, and Early Infant Diagnosis (EID) clinic at LRRH are sent to the cervical cancer screening unit. Screening is conducted mainly by nurses and midwives and rarely by a medical officer. Other cervical screening opportunities in Lira City are one by way of outreaches, which are either part of the hospital program or supported by nongovernment organizations.

### 2.2. Study Participants

The study participants comprised Healthcare providers who offered care to HIV-positive women at LRRH, Lira City. In this study, healthcare providers with experience in cervical cancer prevention and control, and those who provided care to HIV-positive women at their clinics were included for KIIs. Specifically, nurses, midwives, and medical officers who were at the forefront of the implementation of cervical cancer screening were included in the study. Participants were most likely to have been working in antenatal, EID, family planning, and cervical cancer screening clinics. We did not recruit healthcare providers who do not interact with HIV-positive clients for a long time, such as laboratory technicians and sonographers.

### 2.3. Sample Size and Sampling Procedure

We purposely selected LRRH because it is a regional referral hospital with the capacity to conduct cervical cancer screening, and by the fact that it serves a large number of HIV-positive clients at its clinics. Within the hospital, we purposively selected four units/clinics, that is, antenatal, EID, family planning, and cervical cancer screening clinics, because they had healthcare providers with practical experience in cervical cancer screening as well as were more likely to interface with HIV-positive women during their routine clinic days. Thus, healthcare providers with experience in cervical cancer prevention and control and those who provided care to HIV-positive women at their clinics were recruited and included for KIIs. From each clinic/unit, we ensured that a ward manager was included as a participant and medical officers who were attached to the obstetrics and gynaecology department. Ward managers were identified through hospital administrative staff who provided contact details and introduced the research team to the ward managers. Upon introduction, the research assistants purposively recruited current ward managers or their assistants, medical officers, and other healthcare providers into the study, taking into consideration that they had spent at least 6 months in their working stations. Participants were recruited from the wards and interviewed until saturation was reached. After conducting 12 interviews, no new themes were obtained from the data, which marked data saturation. Hence, the final sample size for this study was 12 participants.

### 2.4. Data Collection Tool and Procedures

We used KII guides to collect data from the healthcare providers. The interview guide contained questions in the English language on the knowledge, practices, and perceptions of healthcare providers towards CCS among HIV-positive women (Table 1). The set of questions in this study was adapted from previous studies [28]. The tool was developed and agreed upon by all the researchers and later pretested among healthcare providers at Lira University Teaching Hospital. Following the pretesting, we made adjustments to ensure that the responses would capture the intended themes. The first, second, and third authors trained the research assistants about the tool and the data collection process. Our study was reviewed and given ethical clearance by the Lira University Research Ethics Committee (LUREC). The director of LRRH and the ward managers of the selected wards permitted us to conduct the study at the site. Before data collection, participants were purposively selected and approached individually by the researchers and given appointment dates and desired times for the interviews. Interviews were conducted by two research assistants who held Bachelor's degrees in Midwifery and Public Health. Participants were informed about the purpose of the study, and signed consent forms before the interviews were held. All the interviews were audio-recorded and conducted in private clinical rooms without interference. To ensure rigor, interview questions were openly discussed with the participants using appropriate probes, and the interviewer re-echoed some of the participant responses to ensure clarity. In addition, each interview was conducted by both research assistants, as one would ask/rephrase the questions while the other would record and take notes.

### 2.5. Data Analysis

The audio-recorded interviews were transcribed verbatim by two authors, and transcripts were proofread by the principal investigator to ensure correctness and completeness. The authors divided themselves into two groups and independently came up with codes, which were later discussed and agreed upon by all the authors. Thematic content analysis was used in data analysis, and all the step-by-step approaches were followed [29]. Repetitive phrases and responses during analysis represented the codes. The coding and organization of the categories were supported by the NVivo (QSR International) software. The codes were later reorganized into related groups, which enabled us to formulate the subthemes and the themes. Most of the themes in this study were directly related to the study objectives. Verbal extracts of the participant responses were used to validate our study findings.

### 2.6. Ethical Considerations

The study was approved by the LUREC (LUREC-2022-5). Further approval was gained from the director of LRRH, and the ward managers of the clinics from where participants were recruited. The participants were informed about the study purpose and procedures to gain their consent. All participants consented to audio-recording and full participation in the study by signing written informed consent forms in English. Participants were identified by unique codes instead of real names.

## 3. Results

### 3.1. Characteristics of the Participants

Table 2 describes the key characteristics of the 12 participants. Eight of the participants were in the age group between 25 and 35 years, and four were above 36 years. There were more females [9] than men [3], and slightly more participants had years of experience of less than 10 years [7]. Most participants [7] were diploma holders, and there were more enrolled midwives than the rest of the cadres (Table 2**).**

### 3.2. Emergent Themes

From the research findings, we organized the data into three thematic areas. The themes that emerged include (I) knowledge about cervical cancer and screening, (II) practices of healthcare providers towards CCS among HIV-positive women, and (III) perceptions of healthcare providers on CCS among HIV-positive women. Healthcare providers' perceptions of CCS among HIV-positive women have been distinctively organized into subthemes and sub-subthemes. (Table 3).

### 3.3. Theme 1: Knowledge About Cervical Cancer and Screening

Interviews were conducted with the study participants regarding their knowledge of cervical cancer, which included causes, signs and symptoms, and diagnosis. In addition, the theme explored healthcare providers' understanding of the risk factors of cervical cancer.

#### 3.3.1. Causes of Cervical Cancer

Almost all participants knew about cervical cancer and its cause, whereas the rest stated that the cause is unknown. In addition, some of the participants could not differentiate between the causes and risk factors for cervical cancer.

A participant said

*“It is caused by persistent infection of HPV, and predisposing factors are smoking, multiple sexual partners.” Participant 8, Female.*

#### 3.3.2. Risk Factors of Cervical Cancer

During the interviews about cervical cancer risk factors, it was revealed that most of the participants knew any of the risk factors for cervical cancer among women. Nearly all the participants mentioned multiple sexual partners as one of the risk factors.

A participant said

*“Okay, one is the virus, and also poor hygiene, which results in UTIs. And also producing many children as well as having many partners.” Participant 3, Female.*

#### 3.3.3. Signs and Symptoms of Cervical Cancer

Most participants knew the signs and symptoms of cervical cancer and cited lower abdominal pain, abnormal and irregular vaginal bleeding, and dyspareunia.

*“There can be complaints of lower abdominal pain, irregular bleeding, and maybe pain during sexual intercourse.” Participant 4, Female.*

#### 3.3.4. Diagnosis for Cervical Cancer

Diagnosis for cervical cancer can be done through HPV DNA testing, Pap smear, visual inspection with acetic acid (VIA), and biopsy. All the participants at least knew one method for cervical cancer screening as a way to diagnose cervical cancer. Noteworthy, VIA and HPV testing were the most mentioned methods.

*“Here we do HPV for the virus and VIA for lesions.” Participant 9, Female.*

*“This is done through inserting vaginal spectrum and after removing the sample to take to cancer institute for confirmatory.” Participant 3, Female.*

### 3.4. Theme 2: Practices of Healthcare Providers Towards CCS Among HIV-Positive Women

#### 3.4.1. Services Provided to HIV-Positive Women

Some of the services healthcare providers offer to HIV-positive clients during routine clinic days include health education, STI screening, cervical cancer screening, family planning services, and antiretroviral therapy.

*“Once we give them ARVs, we do cancer screening, family planning, and also health education talks.” Participant 7, Female.*

*“Ah, we offer health education on diseases, cervical cancer screening for those who are eligible, STI screening, and family planning.” Participant 12, Female.*

On the other hand, during health education, the participants reported that they often teach their participants about viral load testing, the importance of screening for cervical cancer, drug adherence, healthy diet, and family planning. This is always done mostly during the routine ART clinic visits.

*“One is on nutrition because you cannot take drugs without food, another one is about family planning and then also talking about cervical cancer screening.” Participant 5, Female.*

*“The common topic is viral load, then adherence, family planning, and many more risky behaviours such as smoking.” Participant 11, Male.*

*“…these talks are done on a daily routine.” Participant 8, Female.*

Also, regarding the cervical cancer preventive services offered at the facility, participants reported that they provide cervical cancer screening, HPV vaccination for eligible girls, and cervical cancer awareness.

*“Vaccination of young girls with the HPV vaccine, cervical cancer screening, and also health education talks.” Participant 7, Female.*

In addition, HPV testing and VIA were the most reported cervical cancer screening modalities.

*“Ah, we have HPV self-testing and also VIA.” Participant 4, Female.*

### 3.5. Theme 3: Perceptions of Healthcare Providers on CCS Among HIV-Positive Women

#### 3.5.1. Impression About the Utilization of Cervical Cancer Screening Services by Women

##### 3.5.1.1. Turn Up for CCS Services

Reportedly, the uptake and demand for cervical cancer screening services by HIV-positive women have notably improved due to healthcare provider recommendations, increased awareness, and continuous appointments for CCS services.

*“Here at the referral, the turn up is good and also the demand is high since they are always reminded, and many referrals are made here.” Participant 8, Female.*

##### 3.5.1.2. Awareness of CCS Services

Most of the participants thought that HIV-positive women are aware of the CCS services, which have led to improved turn up for screening. It is also a result of the routine health education talks provided to these women at the clinics.

*“They are aware due to daily reminders given.” Participant 8, Female.*

##### 3.5.1.3. Barriers and Facilitators for CCS Utilization by HIV-Positive Women

Participants also cited frustrations and hindrances that deter HIV-positive women from seeking and utilizing cervical cancer services at the health facility. Some of the barriers included long waiting hours, long distances to the facility, and the perceived negative attitude of some health providers that provide these services.

*“I think many are discouraged more so if the turn-up is big and they have to wait for many hours to be attended to.” Participant 12, Female.*

*“I think long distances to the health facility and maybe the attitude some have on health providers that most of them are rude.” Participant 5, Female.*

Regardless of the different barriers HIV-positive women face while seeking CCS, it was noted that continuous awareness creation through health education, the presence of signs and symptoms, and the desire to stay healthy motivate them to use these services.

*“First of all, the awareness given to them makes them come, and then also they know that they are supposed to test based on their conditions they have.” Participant 7, Female.*

*“Most of them come after getting the symptoms, so they rush for checkups and treatment.” Participant 9, Female.*

*“My observation is that I think women nowadays are concerned about their lives since they are aware of dangers that may result if not screened.” Participant 12, Female.*

##### 3.5.1.4. Suggestions on Ways that CCS Utilization Can Be Improved

As suggested by most of the participants, CCS can be improved through continuous health education on cervical cancer, community sensitization, and ensuring privacy for the women who go for screening services at the health facility.

*“Maybe intensifying health education talks, and also educating the community more about the dangers of not screening for cervical cancer.” Participant 9, Female.*

*“I think they can put more rooms for privacy….” Participant 3, Female.*

*“I think this could be through Continuous Health Education and sensitization of the community about the benefits of cervical cancer screening services.” Participant 4, Female.*

## 4. Discussion

Our study provides deeper evidence of the knowledge, practices, and perceptions of the healthcare providers on cervical cancer screening among HIV-positive women, the perceived extent of CCS utilization among HIV-positive women, and possible solutions to CCS utilization improvement.

Participants in our study were knowledgeable about cervical cancer and cervical cancer screening, which was expressed in terms of causes, risk factors, signs and symptoms, as well as diagnostic methods of cervical cancer among women. Study participants knew persistent HPV infection as the cause of cervical cancer, knew multiple sexual partners as one of the risk factors, knew abnormal and irregular vaginal bleeding and dyspareunia as the signs and symptoms, and mentioned VIA and HPV testing as the cervical cancer diagnostic methods. Our findings are consistent with those obtained from Northern Uganda [30] and Nigeria [21] and disagree with those obtained among healthcare providers in Saudi Arabia [31]. However, only a few had misconceptions about the signs and symptoms of cervical cancer. The findings imply that the healthcare providers are making substantial contributions concerning patient education on cervical cancer and cervical cancer screening, which could have led to improvement in CCS utilization [26]. The good knowledge of the participants in this study could have been due to prior cervical cancer training at the facility, which could have boosted their understanding of cervical cancer. Since nurses, midwives, and medical officers are always interacting with HIV-positive women, they must have current knowledge about cervical cancer to disseminate accurate information. Timely refresher pieces of training and the provision of updated guidelines on cervical cancer screening would positively impact the knowledge levels of healthcare providers.

Our results demonstrate the different services healthcare providers offer to HIV-positive women during the routine clinic days and at outreaches. Most of the participants reported providing health education, STI screening, cervical cancer screening, family planning services, and antiretroviral therapy to HIV-positive women. Additionally, health education provided by healthcare providers entails the need for viral load testing, the importance of screening for cervical cancer, drug adherence, a healthy diet, and family planning on a routine basis. Prior studies have shown how the provision of health education translates into improved service utilization of CCS services [32, 33]. Hence, there is a need to strengthen continuous education strategies such as health talks on every clinic day in ART clinics, conducting awareness campaigns about cervical cancer screening and individualized health talks to clients to improve CCS utilization. Overall, our findings show that HIV-positive women receive a variety of services from the facility, but it is not clear whether all those services are obtained from the same clinic. This highlights the need to always link HIV-positive women to specific clinics where those services can be obtained through seamless referrals. Alternatively, these services can be integrated into one clinic and provided as a package to reduce missing out on some of the services or rather decentralize the CCS services in various clinics to improve accessibility.

Cervical cancer prevention services commonly provided by healthcare workers included cervical cancer screening, HPV vaccination for eligible girls and cervical cancer awareness. Specifically, HPV testing and VIA were the most reported cervical cancer screening modalities. Notably, cancer prevention by HPV vaccination among eligible girls is still low [24, 34] in Northern Uganda despite improved CCS utilization and cervical cancer awareness in the region [14, 26, 35]. Baseline screening using HPV DNA testing and VIA is an effective way for triage and detection of abnormal cervical lesions among women in low- and middle-income countries [13, 36]. Thus, these screening tests can be adopted at all levels of healthcare in Uganda to increase CCS coverage. This will have implications on the timely detection and treatment of precancerous lesions among women.

Based on our findings, participants reported increasing turn-up and demand for cervical cancer screening services among HIV-positive women. This could be attributed to healthcare provider recommendations [37, 38], increased awareness [26, 39], and continuous appointments for CCS [37] services at the facility. Repetitive reminders and referrals significantly create awareness about the services, which not only facilitates patient timeliness but also efficient utilization of the available services [40]. Our findings indicate an increasing uptake of CCS, which could be a result of the efforts by the health sector and its partners in the region. It also implies the need to increase and create more opportunities for screening among eligible women, both at the facility and community levels. There is a need to adopt more effective and efficient cervical cancer screening techniques, such as self-sample collection for HPV DNA testing, VIA, and referral for treatment of any detected abnormal cervical lesions at all levels.

Our study suggests that HIV-positive women are more likely to undergo cervical cancer screening due to continuous awareness creation through health education, the presence of signs and symptoms, and the desire to maintain good health. Previous studies are in agreement with our findings [41–43]. The findings imply that some women only screen following the onset of symptoms, which may be detected when the disease has already advanced. Moreover, the high mortality rate due to cervical cancer is attributed to the diagnosis of cervical cancer during the late stage, where treatment is impossible [12]. Although existing health education interventions educate the public on notable signs and symptoms of cervical cancer, women should also be encouraged to go for screening even in the absence of symptoms.

Nevertheless, cervical cancer screening is reportedly hindered by long waiting hours, long distances to the facility, and the perceived negative attitude of some health providers that provide these services. This is consistent with results from previous studies in Uganda [43, 44]. The findings imply that the available CCS services are unsatisfactory since they are not easily accessible, as well as due to the delays while seeking those services, thus poor quality care. As a result, service utilization will be negatively impacted due to poor quality of care [45]. Therefore, there is a need to decentralize cervical cancer screening services and adopt and encourage self-cervical cancer screening in low-resource settings. This will help decongest the main screening centres and improve accessibility. Additionally, continuous education about the CCS procedure and its benefits can help address the community's misconceptions and concerns towards CCS and hence foster a need to utilize these services among women.

To enhance the usage of cervical cancer screening services among HIV-positive women, participants were provided with an opportunity to propose effective methods for the implementation of these services. This exercise aimed to gather valuable insights from individuals with relevant experience or knowledge, which could be utilized in designing and executing targeted interventions that could increase the uptake of cervical cancer screening among this specific population. They suggested that CCS can be improved through continuous health education on cervical cancer, community sensitization, and ensuring privacy for the women who go for screening services at the health facility. Therefore, CCS utilization among HIV-positive women can be improved if the clients are well informed and when these services are provided respectfully. By considering the suggestions, it is hoped that healthcare providers and policymakers can create more tailored and effective strategies that address the unique challenges and barriers faced by HIV-positive women when accessing cervical cancer screening services.

### 4.1. Study Limitations

Like all qualitative studies, our study used a small sample size; hence, our findings may not be generalizable to more extensive settings but rather transferable to similar settings. Secondly, we did not include community healthcare providers who are nearer to the community and could have unique perceptions and practices. There could also have been social desirability due to self-reported practices, which was minimized by assuring participants of their confidentiality. This study only focused on the perspectives of healthcare providers at LRRH, which may not reflect the situation of other hospitals in Uganda. However, the findings generated by this study can be transferable to other similar settings.

## 5. Conclusion and Recommendations

Whereas the participants' knowledge and perceptions about CCS were good, their practices towards CCS among HIV-positive women were suboptimal. Continuous education of healthcare providers, decentralization of CCS, and routine and ongoing health education initiatives are pivotal in improving CCS practices among HIV-positive women. There is also an urgent need to strengthen health systems to address the specific barriers to CCS among HIV-positive women and leverage the facilitators to improve CCS utilization.

## Figures and Tables

**Table 1:** Data collection tool.

**S/N**	**Interview question**	**Probe**
1	Please tell me about yourself (age in completed years, level of education, level of training, years of experience, and cadre)	

2	Tell me about cervical cancer and HIV/AIDS among women.	✓What do you know about cervical cancer? (causes, signs, and symptoms, diagnosis) ✓How do you perceive cervical cancer among HIV women? (risk perception)

3	Tell me more about the kind of health education talks you provide to women with HIV.	✓What do you include in the education package? ✓How often are these educational talks provided?

4	Please discuss some of the services you provide to HIV-positive women.	✓Which services are provided to HIV-positive women? ✓Which cervical cancer preventive services are provided at the facility?

5	Please discuss what you understand by cervical cancer screening.	✓Tell me more about its importance ✓Discuss some of the cervical cancer screening modalities provided at the facility.

6	Tell me about your impression of the utilization of cervical cancer screening services by HIV-positive women	✓Tell me about the turn-up and demand for CCS by HIV-positive clients ✓Awareness about cervical cancer screening among HIV-positive women ✓Some of the reasons why some women may or may not use the services ✓Circumstances under which HIV-positive women may use CCS.

7	Please provide any suggestions on ways that screening utilization can be improved	

*Note:* Key informant interview guide subthemes of the knowledge, practices, and perceptions of health care providers on CCS among HIV-positive women in Lira City.

**Table 2:** Characteristics of the participants.

**Variable (** **n** = 12**)**	**Category**	**Frequency (** **n** = 12**)**	**Percentage (%)**
Age	25–35 years	8	66.6
	36–50 years	2	16.7
	Above 50 years	2	16.7

Sex	Female	9	75.0
	Male	3	25.0

Years of experience	<10 years	7	58.3
	Above 10 years	5	41.7

Level of education	Certificate	3	25.0
	Diploma	7	58.3
	Bachelor's degree	2	16.7

Cadre	Enrolled midwife	5	41.6
	Enrolled nurse	3	25.0
	Assistant nursing officer	2	16.7
	Medical officer	2	16.7

*Note:* Characteristics of the healthcare providers in Lira City, Northern Uganda.

**Table 3:** Summary of the themes and subthemes.

**Themes**	**Subthemes**	**Sub subthemes**
Knowledge about cervical cancer and screening	a) Causes of cervical cancer (HPV infection, multiple sexual partners) b) Risk factors of cervical cancer (smoking, multiple sexual partners) c) Signs and symptoms of cervical cancer (lower abdominal pain, abnormal and irregular vaginal bleeding, and dyspareunia) d) Diagnosis of cervical cancer (HPV testing, VIA, Pap smear)	

Practices of healthcare providers towards CCS among HIV positive women.	Services provided to HIV-positive women (health education, STI screening, cervical cancer screening, family planning services, and antiretroviral therapy)	

Perceptions of healthcare providers on CCS among HIV-positive women	Impression about the utilization of cervical cancer screening services by women (utilization of CCS, facilitators and barriers to CCS, and how CCS utilization can be improved)	a) Turn up for CCS services b) Awareness of CCS services c) Barriers and facilitators for CCS utilization by HIV-positive women d) Suggestions on ways that CCS utilization can be improved

*Note:* Summary of the themes, subthemes, and sub-subthemes of the knowledge, practices, and perceptions of health care providers on CCS among HIV-positive women in Lira City.

## Data Availability

All relevant materials and methods concerning the study will be made available on request.
